# Changing the Name of Diabetes Insipidus: A Position Statement of the Working Group to Consider Renaming Diabetes Insipidus

**DOI:** 10.1159/000527139

**Published:** 2022-09-30

**Authors:** Hiroshi Arima, Timothy Cheetham, Mirjam Christ-Crain, Deborah L. Cooper, Juliana B. Drummond, Mark Gurnell, Miles Levy, Ann McCormack, John D. Newell-Price, Joseph G. Verbalis, John Wass

**Affiliations:** ^a^Department of Endocrinology and Diabetes, Nagoya University Graduate School of Medicine, Nagoya, Japan; ^b^Newcastle University, Newcastle upon Tyne, UK; ^c^Great North Children's Hospital (GNCH), Newcastle upon Tyne, UK; ^d^Department of Endocrinology, University Hospital and University of Basel, Basel, Switzerland; ^e^Pituitary Foundation, Bristol, UK; ^f^Department of Internal Medicine, School of Medicine, Federal University of Minas Gerais, Belo Horizonte, Brazil; ^g^Wellcome-MRC Institute of Metabolic Science, University of Cambridge and Addenbrooke's Hospital, Cambridge Biomedical Campus, Cambridge, UK; ^h^Department of Endocrinology, University Hospitals of Leicester NHS Trust, Leicester, UK; ^i^Department of Endocrinology, St Vincent's Hospital, Sydney, New South Wales, Australia; ^j^Garvan Institute of Medical Research, Sydney, New South Wales, Australia; ^k^St Vincent's Clinical School, University of New South Wales, Sydney, New South Wales, Australia; ^l^Department of Oncology and Metabolism, The Medical School, University of Sheffield, Sheffield, UK; ^m^Georgetown-Howard Universities Center for Clinical and Translational Science, Georgetown University, Washington, District of Columbia, USA; ^n^Department of Endocrinology, University of Oxford, Churchill Hospital, Oxford, UK

“*What's in a name? That which we call a rose / By any other name would smell as sweet.*” (Juliet, from *Romeo and Juliet* by William Shakespeare). Shakespeare's implication is that a name is nothing but a word and it therefore represents a convention with no intrinsic meaning. Whilst this may be relevant to romantic literature, disease names do have real meanings, and consequences, in medicine. Hence, there must be a very good rationale for changing the name of a disease that has a centuries-old historical context. A working group of representatives from national and international endocrinology, and pediatric societies now proposes changing the name of “diabetes insipidus” to “arginine vasopressin deficiency (AVP-D)” for central etiologies, and “arginine vasopressin resistance (AVP-R)” for nephrogenic etiologies. This editorial provides both the historical context and the rationale for this proposed name change.

## Reasons for Changing a Disease Name

Understanding of disease processes is a dynamic field, with rapidly evolving concepts of pathophysiology based on emerging molecular and genetic data. Consequently, newer understanding of pathophysiology is one of the major reasons for renaming diseases. In endocrinology, appreciation of hyperprolactinemia as the common pathophysiology underlying many different clinical situations causing galactorrhea and amenorrhea led to the effective abandonment of many previous eponymous names for these conditions such as Chiari-Frommel syndrome, Forbes-Albright syndrome, Ahumada-del Castillo syndrome, etc. [1]. A second reason is based on historical discoveries that a previous eponymous name for a syndrome was inappropriately attributed to an individual who was not the first or even the most significant person involved in the description of the syndrome [2]. A third reason is later appreciation of medically unethical behaviors of individuals with diseases eponymously named for them, as characterized by the renaming of Reiter's syndrome to “reactive arthritis” and Wegener's granulomatosis to “granulomatosis with polyangiitis,” because of the association of the eponymous physicians with Nazi antihumanitarian crimes [3, 4]. The first three of these reasons for changing disease names make a strong case for detaching eponyms from disease processes whenever possible [5]. However, endocrinologists would be loath to abandon the eponyms of Addison, Cushing, Hashimoto and others for their unique and seminal contributions to our understanding of endocrine disease processes. However, yet a fourth reason for renaming diseases is when traditional disease names lead to confusion between pathophysiologically different processes, leading to treatment errors and consequent adverse outcomes for patients. This latter reason represents the major impetus to change the name of diabetes insipidus at this time.

## Historical Context

Before explaining the rationale for the name change, it is instructive to review the historical context for the name of diabetes insipidus. The polyuria and polydipsia of diabetes was first described by Demetrius of Apameia (1st–2nd century BC), who used the term “diabetes,” meaning “passing water like a siphon” to describe the polyuria characteristic of this condition. Araetus of Cappadocia (81–138 AD) further defined the clinical characteristics of this disease [6]. Although observations that the urine was sweet were alluded to in both Greek and Indian history, the first documented report of the sweet character of diabetic urine was published by the English physician Sir Thomas Willis in 1674 (“The Diabetes or Pissing Evil”). However, the differentiation between the saccharine urine of glucosuria and the non-saccharine urine of other forms of polyuria is attributed to the Scottish physician William Cullen, who appended the Latin word “mellitus” (sweet) to the Greek term diabetes to distinguish between these two types of polyuria [7]. In 1794, Johann Peter Frank first introduced the term “diabetes insipidus” to differentiate these patients from those with diabetes mellitus [7]. These terms persisted as valid clinical descriptions without known pathophysiology until the vasopressor and antidiuretic actions of posterior pituitary extracts were discovered in the late 19th and early 20th century, including use of posterior pituitary extracts to treat diabetes insipidus. In the mid-20th century, arginine vasopressin was synthesized and identified as the antidiuretic hormone, and the distinct central and nephrogenic etiologies of diabetes insipidus were recognized and characterized [8]. Despite new knowledge of the underlying pathophysiology of the different etiologies of diabetes insipidus by the late 20th century, no attempts were made to rename diabetes insipidus according to the known causes of the disorder, namely deficiency of arginine vasopressin or resistance to the receptor-mediated actions of arginine vasopressin.

## Rationale for Changing the Name of Diabetes Insipidus

There are multiple reasons to change the name of diabetes insipidus at this time. First and foremost, although the terms mellitus and insipidus do differentiate between the clinical characteristics of these two very different causes of polyuria, and clearly are not eponyms, the use of the common term “diabetes” in both has unfortunately led to confusion for both patients and their caretakers. This confusion with diabetes mellitus has been to the detriment of patients with diabetes insipidus when they are under the care of non-endocrine specialists. Some physicians and nurses do not appreciate the difference between these two very different disorders. In several patients with central diabetes insipidus, desmopressin treatment was withheld with serious adverse outcomes, including death [9]. This has led to high profile litigation cases and coroners' inquests involving the police, with wide media coverage. Subsequent to these unfortunate but avoidable cases, national safety alerts, surveys amongst endocrinologists, and a global task-force consisting of a wide range of senior clinicians involved with the care of patients with diabetes insipidus has led to a strong impetus to change the name of the condition. Second, patients with diabetes insipidus strongly support changing the name to eliminate “diabetes.” In a survey of >1,000 patients with central diabetes insipidus recently published in *The Lancet Diabetes & Endocrinology* [10], 85% preferred the name to be changed, mainly because of experiences with insufficient understanding of the disease by health professionals who confused this disorder with diabetes mellitus. Eighty-seven percent of patients felt that this lack of knowledge and the resulting clinical confusion affected the management of their condition, e.g., repeated blood sugar measurements or prescription of medication for diabetes mellitus during hospitalization. Finally, we believe the names of medical disorders optimally should reflect the underlying pathophysiology, which in the case of diabetes insipidus is now well known to be deficient secretion and/or end-organ effects of the hormone arginine vasopressin (AVP). Hence, for all the above reasons, the working group proposes that the name diabetes insipidus should be changed to “arginine vasopressin deficiency (AVP-D)” for central etiologies, and “arginine vasopressin resistance (AVP-R)” for nephrogenic etiologies, and this proposal has been endorsed by the following societies represented by the working group members: Endocrine Society, European Society of Endocrinology, Pituitary Society, Society for Endocrinology, European Society for Paediatric Endocrinology, Endocrine Society of Australia, Brazilian Endocrine Society, and Japanese Endocrine Society, and is under review at several other societies.

## Implementation of the Name Change for Diabetes Insipidus

In order to ease the transition in terms of on-line searches and avoid confusion in the literature, we propose that for several years we keep the previous name in parentheses. Therefore, we will begin using the terms AVP-deficiency (cranial or central diabetes insipidus) and AVP-resistance (nephrogenic diabetes insipidus) in manuscripts and chapters. Once the transition is complete, it is likely that the parenthetic term will be lost, albeit people can still use it if they wish. In addition, we have initiated a request to the ICD Coordination and Maintenance Committee to have the ICD-11 coding changed to reflect the new names.

We fully recognize that changing a name for a long-standing disease is never easy. But just as the rheumatologists who proposed the name change of granulomatosis with polyangiits (Wegener's granulomatosis) [4], we hope our medical colleagues will recognize and accept the above rationale for making this change, both in the interest of scientific accuracy, but more so for the benefit and safety of our mutual patients with diabetes insipidus so that their disease and its treatment will no longer be confused with diabetes mellitus.

## Conflict of Interest Statement

The authors have no conflicts of interest to declare.

## Funding Sources

This paper did not receive any funding.

## Author Contributions

All authors wrote and reviewed the position statement.
